# Autophagic Cell Death During Development – Ancient and Mysterious

**DOI:** 10.3389/fcell.2021.656370

**Published:** 2021-04-09

**Authors:** Lawrence M. Schwartz

**Affiliations:** Department of Biology, Molecular and Cellular Biology Program, Morrill Science Center, University of Massachusetts, Amherst, MA, United States

**Keywords:** programmed cell death, apoptosis, autophagy, autosis, necrosis, Tat-Beclin 1, lysosome

## Abstract

While cell death is a normal and essential component of development and homeostasis, dysregulation of this process underlies most human diseases, including cancer, autoimmunity and neurodegeneration. The best characterized mechanism for cell death is apoptosis, although some cells die by a distinct process known as autophagy-dependent cell death (ADCD). Autophagy is mediated by the formation of double membrane vesicles that contain protein aggregates, damaged organelles like mitochondria, and bulk cytoplasm, which then fuse with lysosomes to degrade and recycle their contents. Autophagy is typically viewed as an adaptive process that allows cells to survive stresses like nutrient deprivation, although increasing evidence suggests that it may also mediate cell death during development and pathogenesis. An aggressive form of autophagy termed autosis has been described in cells following either ischemia/reperfusion injury or in response to autophagy-inducing proteins like Tat-Beclin 1. Despite an extensive literature on autophagic cell death in a variety of contexts, there are still fundamental gaps in our understanding of this process. As examples: Does autophagy directly kill cells and if so how? Is ADCD activated concurrently when cells are triggered to die via apoptosis? And is ADCD essentially a more protracted version of autosis or a distinct pathway? The goal of this mini-review is to summarize the field and to identify some of the major gaps in our knowledge. Understanding the molecular mechanisms that mediate ADCD will not only provide new insights into development, they may facilitate the creation of better tools for both the diagnostics and treatment of disease.

## Introduction

The term programmed cell death (PCD) was coined by Lockshin and Williams (1965) to describe the precisely timed loss of skeletal muscles at the end of metamorphosis in moths. PCD plays many essential roles during both development and homeostasis. First, it can insure the presence of the appropriate number of cells within each tissue. A general rule of embryogenesis is that many more cells are produced in each lineage than are ultimately needed to support organogenesis (Raff, 1992). Cells that make a connection with appropriate partners receive a retrograde signal referred to as a trophic factor. These cells then upregulate survival programs and persist, while their unsuccessful neighbors die during a discrete window of development. The classical example of this process is the matching of motor neurons to the muscles they innervate (Hamburger, 1934). Second, targeted cell death can help sculpt the body, such as interdigital cell death in the developing limb bud to form the fingers (Saunders et al., 1962). Third, cell death can target tissues that have served an important function at one stage of development but are then no longer needed at a later stage, such as regression of the tadpole tail during amphibian metamorphosis (Smith and Tata, 1976). These “surplus” tissues represent valuable reservoirs of macromolecules that can be used to support metabolism and development. Fourth, PCD can be used to remove deleterious cells, such as the loss of self-reactive thymocytes during negative selection in the thymus (Surh and Sprent, 1994). Lastly, PCD functions as a normal component of homeostasis. For example, we lose approximately one million cells per minute throughout life, primarily in the hematopoietic system (Levine and Ucker, 2019). These cells are typically replaced by the products of stem cells, thus maintaining tissue homeostasis.

However, not all cell deaths serve a constructive role; it has been estimated that misregulation of this process may account for upwards of 70% of human disease (Reed, 2002). In some cases, inappropriate activation of PCD results in the loss of valuable and irreplaceable cells, thus compromising the tissue. This is the basis of essentially all neurodegenerative disorders like Alzheimer’s and Parkinson’s diseases. In contrast, the failure to delete defective but mitotically-competent cells allows for their clonal expansion, which serves as the basis of most cancers and all autoimmune diseases. Consequently, one of the major drivers of the field is the desire to identify interventions that rescue valuable but condemned cells, or alternatively, selectively target defective ones.

During development, the molecules or cell-cell interactions that trigger PCD typically are not inherently toxic but instead are physiological signals that can activate a range of downstream responses, one of which may be death. This is illustrated in amphibian metamorphosis, where a dramatic increase in the circulating levels of thyroxine simultaneously induces proliferation and differentiation in the developing limb anlagen and cell death in the tail (Yaoita, 2019). However, cell death can also be provoked by external signals that do not normally function during development, such as ionizing radiation or toxins. Previous debate in the field questioned whether these cell deaths can accurately be termed “programmed,” since they do not occur in a temporally or spatially predictable manner. To address this issue, the term Accidental Cell Death (ACD) was introduced to identify deaths that are induced by exogenous insults, while the term Regulated Cell Death (RCD) was coined to capture those cell deaths that employ specific cellular machinery, independent of the upstream trigger (Galluzzi et al., 2018). Thus, while the loss of immature T cells during negative selection is a traditional example of RCD, the killing of these same cells with ionizing radiation represents ACD, even though they both lead to apoptosis (Lowe et al., 1993; Galluzzi et al., 2018). PCD is still an acceptable term for those deaths that occur as a normal component of development.

## Cell Death Programs

Historically, three distinct cell death programs were recognized based on morphological criteria: type I (nuclear degeneration/apoptosis), type II (cytoplasmic vacuolization/autophagic cell death), and type III degeneration (cell rupture/necrosis) (Schweichel and Merker, 1973; Clarke, 1990) (The term “degeneration” in this context is archaic and not in current use). The intense scientific focus on cell death (∼500,000 citations in Pubmed by the start of 2021) has revealed that there are many more pathways that can mediate cell loss. In addition to the three listed above, other programs include: ferroptosis, pyroptosis, necroptosis, parthanatos, entosis, mitochondrial permeability transition (MPT)-driven necrosis, lysosome-dependent cell death, NETotic cell death, oxytosis, and mitotic catastrophe (reviewed in Galluzzi et al., 2018; Tang et al., 2019; Nirmala and Lopus, 2020).

Necrosis (formerly “type III degeneration”) was initially viewed as a passive process whereby mechanical, chemical, or osmotic insults result in cellular swelling, membrane disruption and subsequent lysis. More recently, several regulated forms of necrosis have been identified, including necroptosis, pyroptosis, parthanoptosis, NETosis, MPT-driven necrosis, oxytosis, and ferroptosis, which are all dependent upon specific signaling pathways within the cell (Degterev et al., 2005; Dixon et al., 2012; Vanden Berghe et al., 2014; Alu et al., 2020). Since cytoplasmic and nuclear constituents are highly inflammatory (collectively known as damage-associated molecular patterns or DAMPS), these deaths can play valuable roles by mobilizing the immune system to respond to an acute injury or infection (Zindel and Kubes, 2020). In contrast, as membrane integrity is maintained during apoptosis, dying cells are typically phagocytosed and degraded by neighboring cells or macrophages, thereby precluding immunological responses. This is obviously beneficial given the massive numbers of cells that die during development and homeostasis, since it would be disastrous for the organism to be in a chronic hyper-inflammatory state. [It should be noted that secondary necrosis of apoptotic cells *in vitro*, which serves as the basis for some widely used cell death assays, including vital dye exclusion, is an artifact resulting from the absence of phagocytic cells (Krysko et al., 2008). The discarded apoptotic bodies run out of the ATP required to maintain membrane pumps and undergo necrosis].

The best understood cell death program is apoptosis (formerly type I degeneration). This is due in part to outstanding genetic models like the nematode *Caenorhabditis elegans*, where the key components of the program were identified via mutational analysis (Malin and Shaham, 2015). In addition, the availability of highly sensitive and reliable assays like TUNEL (DNA fragmentation) and Annexin V staining (phosphatidylserine externalization) allow for the easy detection of apoptotic cells both *in vitro* and *in vivo* (Galluzzi et al., 2009).

Apoptosis is typically driven by the activation of pro-caspases, a family of cysteine-aspartic proteases that cleave a vast range of cellular proteins (Julien and Wells, 2017). There are two main pathways for caspase activation- extrinsic and intrinsic (reviewed in Bedoui et al., 2020). Ligand binding to membrane death receptors like TRAIL and FAS lead to the formation of the Death-Inducing Signaling Complex (DISC), and the subsequent activation of pro-caspase-8, which then activates the downstream executioner pro-caspases-3 and -7. The intrinsic pathway is initiated when developmental or pathological signals lead to a shift in the balance between pro- and anti-apoptotic Bcl-2 family members that regulate the oligomerization of BAX and BAK. These proteins can form a pore that mediates mitochondrial outer membrane permeabilization (MOMP) causing the release of cytochrome *c* and other pro-apoptotic proteins like SMAC/DIABLO and apoptosis inducing factor (AIF) (Kalkavan and Green, 2018). Cytochrome *c* and dATP bind to apoptotic protease activating factor *1* (APAF-1) to facilitate the formation of the apoptosome and the subsequent activation of the initiator caspase-9, and ultimately, the activation of executioner caspases. The extrinsic and intrinsic pathways can be coupled via caspase-8 cleavage of the BH3-only protein Bid to form truncated Bid (tBid) (Li et al., 1998).

**FIGURE 1:**
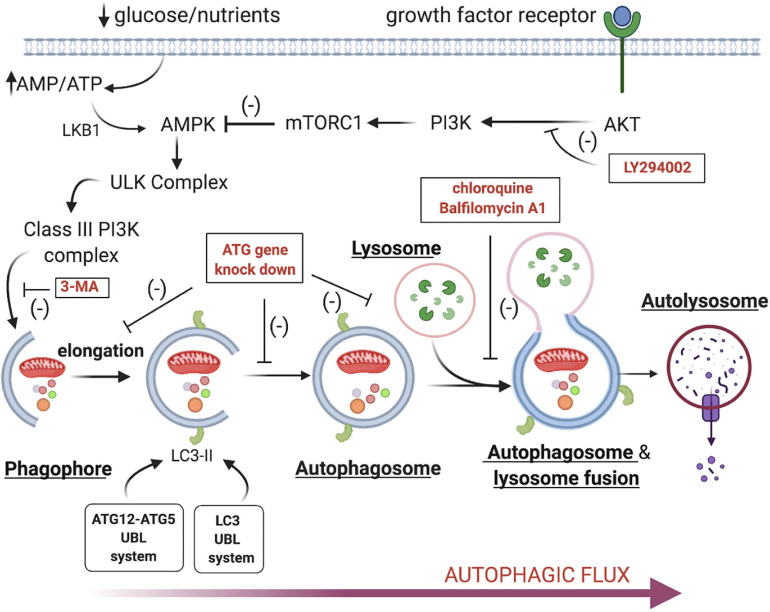
Signal transduction cascade regulating autophagy. Loss of growth factor stimulation or nutritional inputs like glucose lead to the activation of the ULK complex and the subsequent activation of the Class III PI3 Kinase complex. This drives phagophore assembly and the subsequent recruitment of cellular constituents like damaged mitochondria, protein aggregates and bulk cytoplasm. The phagophore elongates and circularizes to form the autophagosome, which then docks with lysosomes. The fusion of these two organelles leads to autolysosome formation and the destruction of the enclosed cargo via lysosomal hydrolases. [It should be noted that this is distinct from lysosome-mediated cell death in which lysosome rupture and release degradative enzymes like cathepsins (reviewed in Wang et al., 2018)]. Several inhibitors or genetic interventions are available that can block autophagy at key regulatory points (labeled red in boxes) (Vakifahmetoglu-Norberg et al., 2015).

Much less is known about the third classic mechanism of cell death- Type II degeneration/autophagic cell death. The best characterized form of autophagy is macroautophagy, and the two terms are often used interchangeably (Other autophagic mechanisms also include microautophagy and chaperone-mediated autophagy). Autophagy is an evolutionarily ancient process that allows cells to survive starvation and other stresses by facilitating the sequestration and degradation of bulk cytoplasm, damaged organelles like mitochondria and protein aggregates (Hughes and Rusten, 2007; Ohsumi, 2014; Wanderoy et al., 2020). The recycled amino acids and fatty acids facilitate ATP production and other essential processes. The basal levels of autophagy can be upregulated under conditions of nutrient deprivation or loss of growth factors (Figure 1; reviewed in Cicchini et al., 2015). This leads to inhibition of mTorc1 and/or the activation of 5’ AMP-activated protein kinase (AMPK) and Class III phosphatidylinositol 3-kinase. These drive the formation of the phagophore, a crescent shaped double membrane structure generated from intracellular membranes. The elongation of the phagophore membrane is mediated by two ubiquitin-like enzymatic cascade (Atg12-Atg5 and LC3/Atg8), which facilitates the phosphatidylethanolamine-lipidated LC3 (ATG8) and it’s recruitment to the membrane (reviewed in Martens and Fracchiolla, 2020). The phagophore then circularizes to form the autophagosome, which then fuses with lysosomes, resulting in the creation of the autolysosomes, where the trapped contents are degraded.

## Autophagy-Dependent Cell Death

Given that autophagy can be an adaptive response that helps cells survive adverse conditions, there is still a lack of clarity of if and how autophagy is part of a directed killing program. In fact, Kroemer and Levine (2008) have suggested that the term “autophagic cell death” is a misnomer and that a more appropriate descriptor might be “cell death with autophagy”. Consequently, in order to determine that cells die via autophagy-dependent cell death (ADCD), it has been proposed that three criteria must be met: (1) other forms of cell death have been excluded; (2) there is an increase in autophagic flux; and (3) genetic or pharmacological blockade of autophagy rescues the cell (Shen and Codogno, 2011; Galluzzi et al., 2018). These strict requirements have been met in only a small number of studies, so the number of bona fide ADCD systems is likely more modest than reflected by a Pubmed search with “autophagic cell death” as the search term (Bialik et al., 2018). Nevertheless, there are several genetic models where an absolute requirement for the autophagic machinery has been shown to be essential for cell death. From a phylogenetic perspective, perhaps the most ancient example is found in the free-living social ameba *Dictyostelium discoideum* (Cornillon et al., 1994). While “*Dicty*” live solitary lives in the soil, the loss of food triggers a signal transduction cascade that induces the cells to aggregate and form a stalk with a spore-containing fruiting body at the top. As differentiation proceeds, the stalk cells die by an autophagy-dependent pathway. Indeed, targeting the autophagy-related gene-1 (*Atg1*) blocks death and the cells instead default to necrosis (Kosta et al., 2004).

The fruit fly *Drosophila melanogaster* also provides several well-characterized examples of autophagic cell death during metamorphosis. The loss of the salivary glands is dependent on both apoptosis and autophagy, as genetic interference with either of these pathways represses but does not prevent cell death (Martin and Baehrecke, 2004; Berry and Baehrecke, 2007). In contrast, the death of the gut appears to require just autophagy, as genetic inactivation or knockdown *Atg1*, *Atg2* or *Atg18* severely delays these deaths (Denton et al., 2009, 2012). Midgut death in flies is recognized as the clearest and most convincing example of ADCD during development.

There are a number of studies supporting the existence of ADCD in mammals, most notably in terminally differentiated cells like muscles and neurons, and some cancers (Linder and Kögel, 2019). Examples include the loss of neurons in neurodegenerative disorders like Alzheimer’s disease (Nixon et al., 2005; Bredesen, 2008), spinal cord injury (Kanno et al., 2011), MPTP-induced cytotoxicity (Zhao et al., 2019), and neonatal hypoxia-ischemia following asphyxia (Ginet et al., 2014). Perhaps the best supported examples have focused on the death of hippocampal stem cells following insulin withdrawal *in vitro* (Yu et al., 2008) or following chronic stress *in vivo* (Jung et al., 2020).

Cells that are null for the pro-apoptotic proteins BAK and BAX, and thus incapable of initiating apoptosis provide intriguing insights into ADCD (Shimizu et al., 2004; Arakawa et al., 2017). When mouse embryonic fibroblasts from *Bax*^–/–^*Bak^–/–^* mice were treated with the potent apoptosis inducers etoposide or staurosporine, they initiated ADCD (Shimizu et al., 2004). Concurrent inactivation of *Atg5* in *Bax^–/–^Bak^–/–^* mice restricted both apoptosis and autophagy. This led to the retention of otherwise condemned cells, including interdigital cells in the limb buds, thymocytes, and some neurons (Arakawa et al., 2017). These data provide strong evidence autophagy is required for PCD during normal mammalian development.

In 2013 a novel form of autophagic cell death termed “autosis” was described in cells that are treated with Tat-Beclin 1, a potent cell-permeant autophagy inducer (Liu et al., 2013; Liu and Levine, 2015). Autotic cells display a dramatic increase in the number of autophagic vesicles and empty vesicles within the cytoplasm. Over time the mitochondria become electron dense, the perinuclear space balloons as the inner and outer nuclear membranes separate, and the nucleus displays concavity. A similar set of morphological changes can be observed *in vivo* in cardiomyocytes in adult mice and rat neonatal neurons following ischemia/reperfusion injury (Liu et al., 2013; Nah et al., 2016, 2020), a process that is clinically relevant to heart attacks and strokes respectively. Autosis can be inhibited genetically by inactivating essential autophagy genes (e.g., *Beclin1* and *Atg7*) or pharmacologically with anti-autophagic drugs that block phagophore assembly (e.g., 3-methyladenine) but not lysosome fusion (e.g., Bafilomycin A1) (Figure 1; Liu et al., 2013). In a high throughput screen, it was also found that inhibitors of Na^+^, K^+^ adenosine triphosphatase (ATPase) like the cardiac glycosides ouabain and digoxin can also inhibit autosis, which is diagnostic for autosis (Liu et al., 2013; Nah et al., 2020).

## Unanswered Questions

While the data presented in this review provides an overview of cell death in general, and ADCD during development in particular, there are still several major gaps in our fundamental understanding of this process (Lindqvist et al., 2015; Bialik et al., 2018). Some of the key questions that remain unresolved are discussed below.

**FIGURE 2:**
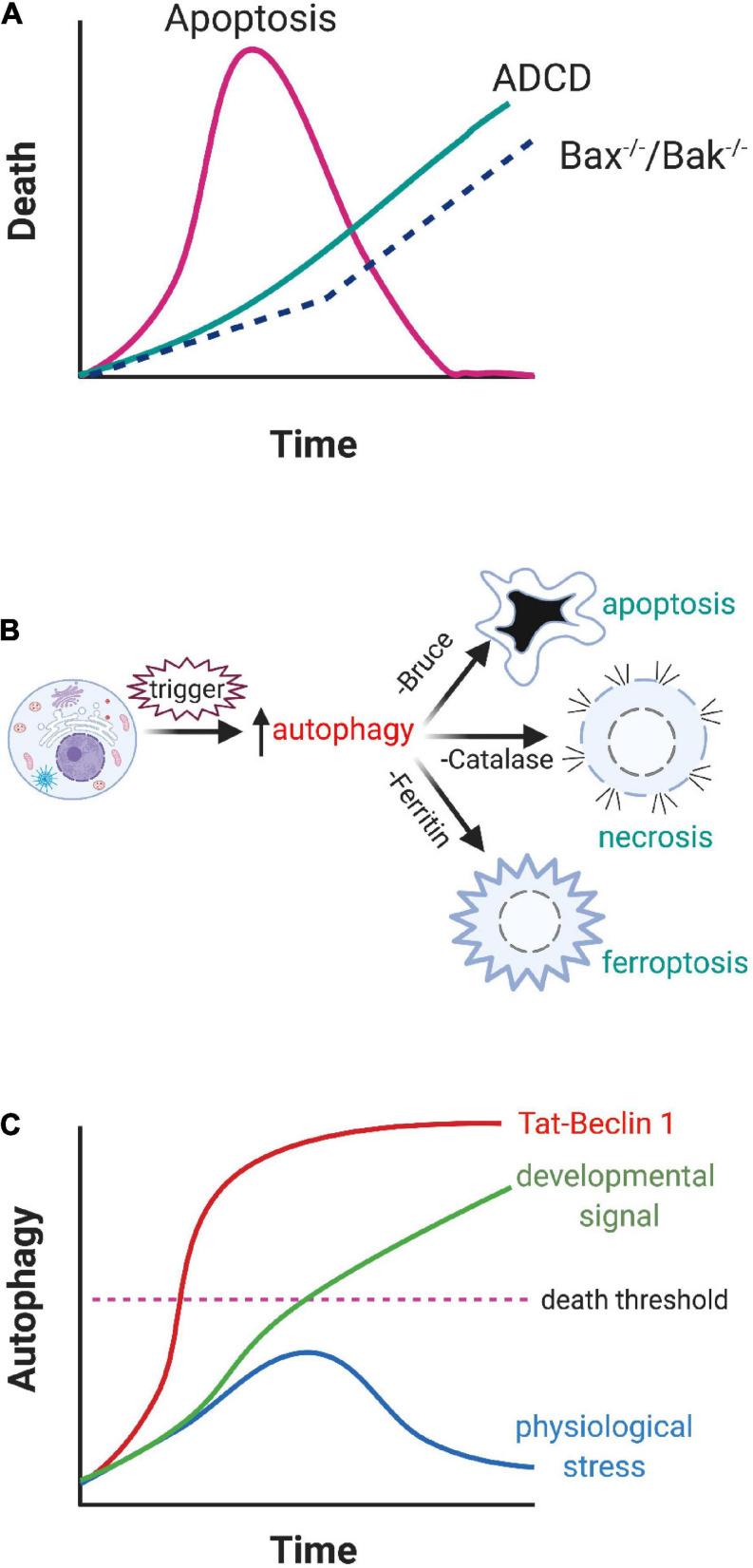
Proposed ADCD pathways. **(A)** Both apoptosis and ADCD are triggered concurrently but the speed of apoptosis masks the involvement of ADCD. Blockage of apoptosis in *Bax^–/–^ Bak^–/–^* cells allows autophagy to progress to the death of the cell. **(B)** Autophagy derepresses other cell death programs. By selectively targeting key survival proteins, compromised cells are able to activate non-autophagic cell death programs. **(C)** Treating cells with potent autophagy inducers like Tat-Beclin 1 results in the rapid demise of the cells (above the dashed “death threshold” line). If a cell is subjected to mild stresses, it transiently upregulates autophagy, which helps it survive the insult. However, when the cell is exposed to a cell death inducer during development, autophagy is driven past the point of no return and the cell dies.

1. Is ADCD widespread but masked by apoptosis?: Some insight into this question is provided from the study of cells from *Bax^–/–^Bak^–/–^* mice, where condemned cells are precluded from undergoing apoptosis and default to ADCD (Shimizu et al., 2004; Arakawa et al., 2017). There are two possible interpretations of these data. The first is that apoptosis and ADCD are triggered concurrently, but ADCD is masked by the much faster apoptosis program (Figure 2A). Alternatively, when cells are unable to activate their primary cell death program, there is compensatory initiation of another one to ensure that condemned cells are removed. Given the complicated cross-talk between autophagic and apoptotic proteins, such as Beclin 1 and Bcl-2 (Xu and Qin, 2019), this process is feasible but challenging to resolve (reviewed in Tsapras and Nezis, 2017).
2. Does autophagy trigger cell death indirectly? Autophagy can selectively target the destruction of key survival proteins like the inhibitor of apoptosis protein (IAP) BRUCE (which prevents pro-caspase activation) (Nezis et al., 2010), catalase (which blocks free radical generation) (Yu et al., 2006), and ferritin (which sequesters iron) (Gao et al., 2016). Loss of these survival proteins subsequently facilitates cell death (Figure 2B). However, instead of dying with an autophagic morphology, these condemned cells die by apoptosis, necrosis, or ferroptosis respectively. Consequently, while autophagy can derepress cell death, it does not meet the three requirements of ADCD in that the actual death process is mediated by a different program.
3. Is ADCD a less aggressive form of autosis? A number of studies have shown a direct correlation between autosis and cell death. Indeed, inhibition of autosis with cardiac glycosides can rescue these otherwise condemned cells (Liu et al., 2013; Nah et al., 2020). One possibility is that autosis is just a more aggressive version of the same program that mediates ADCD during development (Figure 2C). In support of this hypothesis, knockdown of upstream autophagy regulators (*beclin1*, *Atg13*, and *Atg14*) can block Tat-Beclin 1-induced cell death (Liu et al., 2013). An overlap between autosis and ADCD would offer mechanistic insights into the cell death process since autosis is accompanied by damage to organelles like mitochondria and the nucleus (Liu et al., 2013). In addition, the aggressive production of intracellular vacuoles during autosis is associated with the breakdown of the Golgi complex and the endoplasmic reticulum, which depletes intracellular membranes, something that is seen in some examples of ADCD (Liu and Levine, 2015). This may explain why Bafilomycin A1, which blocks lysosome fusion to autophagosomes, does not inhibit autosis (Liu et al., 2013). In the case of ischemia/reperfusion injury there is also a dramatic increase in the production of destructive free radicals (Wu et al., 2018). Collectively, these insults likely bring about the demise of the cell. However the relationship between ADCD and autosis is unclear. All of the examples of autosis described thus far result from pathological drivers of autophagy, like Tat-Beclin 1 or ischemia/reperfusion injury, rather than by physiological changes within a developmental context. It should also be noted that the morphologies associated with ADCD and autosis are distinct, although this may reflect the faster kinetics or intensity of autosis relative to the more protracted time course of ADCD. Clearly this is an area that warrants further investigation.

## Conclusion

While it has not been demonstrated unambiguously that autophagy drives cell death during development (Kroemer and Levine, 2008), there is increasing evidence that ADCD is a bonafide cell death program. Nevertheless, there are still major gaps in our understanding of this mechanism in development and pathogenesis. Resolving these issues will not only advance our fundamental understanding of an ancient cellular process that is relevant to developmental biology and homeostasis, it may provide insights into the diagnostics and/or therapeutics for disease. In particular, ADCD appears to be an important process in neurodegeneration, cancer, and cardiology. The field will benefit greatly from the identification of the kinds of markers and inhibitors that have propelled the study of apoptosis.

## Author Contributions

LS wrote the manuscript and prepared the figures.

## Conflict of Interest

The author declares that the research was conducted in the absence of any commercial or financial relationships that could be construed as a potential conflict of interest.

## References

[B1] Alu A. Han X. Ma X. Wu M. Wei Y. Wei X. (2020). The role of lysosome in regulated necrosis. *Acta Pharm. Sin. B.* 10 1880–1903. 10.1016/j.apsb.2020.07.003 33163342 PMC7606114

[B2] Arakawa S. Tsujioka M. Yoshida T. Tajima-Sakurai H. Nishida Y. Matsuoka Y. (2017). Role of Atg5-dependent cell death in the embryonic development of bax/bak double-knockout mice. *Cell Death Differ.* 24 1598–1608. 10.1038/cdd.2017.84 28574506 PMC5563990

[B3] Bedoui S. Herold M. J. Strasser A. (2020). Emerging connectivity of programmed cell death pathways and its physiological implications. *Nat. Rev. Mol. Cell Biol.* 21 678–695. 10.1038/s41580-020-0270-8 32873928

[B4] Berry D. L. Baehrecke E. H. (2007). Growth arrest and autophagy are required for salivary gland cell degradation in drosophila. *Cell* 131 1137–1148. 10.1016/j.cell.2007.10.048 18083103 PMC2180345

[B5] Bialik S. Dasari S. K. Kimchi A. (2018). Autophagy-dependent cell death - where, how and why a cell eats itself to death. *J. Cell Sci.* 131:jcs215152. 10.1242/jcs.215152 30237248

[B6] Bredesen D. E. (2008). Programmed cell death mechanisms in neurological disease. *Curr. Mol. Med.* 8 173–186. 10.2174/156652408784221315 18473818

[B7] Cicchini M. Karantza V. Xia B. (2015). Molecular pathways: autophagy in cancer–a matter of timing and context. *Clin. Cancer Res.* 21 498–504. 10.1158/1078-0432.CCR-13-2438 25165101 PMC4315744

[B8] Clarke P. G. (1990). Developmental cell death: morphological diversity and multiple mechanisms. *Anat. Embryol.* 181 195–213. 10.1007/BF00174615 2186664

[B9] Cornillon S. Foa C. Davoust J. Buonavista N. Gross J. D. Golstein P. (1994). Programmed cell death in dictyostelium. *J. Cell Sci.* 107 2691–2704. 7876338 10.1242/jcs.107.10.2691

[B10] Degterev A. Huang Z. Boyce M. Li Y. Jagtap P. Mizushima N. (2005). Chemical inhibitor of nonapoptotic cell death with therapeutic potential for ischemic brain injury. *Nat. Chem. Biol.* 1 112–119. 10.1038/nchembio711 16408008

[B11] Denton D. Chang T. K. Nicolson S. Shravage B. Simin R. Baehrecke E. H. (2012). Relationship between growth arrest and autophagy in midgut programmed cell death in drosophila. *Cell Death Differ.* 19 1299–1307. 10.1038/cdd.2012.43 22555456 PMC3392632

[B12] Denton D. Shravage B. Simin R. Mills K. Berry D. L. Baehrecke E. H. (2009). Autophagy, not apoptosis, is essential for midgut cell death in drosophila. *Curr. Biol.* 19 1741–1746. 10.1016/j.cub.2009.08.042 19818615 PMC2783269

[B13] Dixon S. J. Lemberg K. M. Lamprecht M. R. Skouta R. Zaitsev E. M. Gleason C. E. (2012). Ferroptosis: an iron-dependent form of nonapoptotic cell death. *Cell* 149 1060–1072. 10.1016/j.cell.2012.03.042 22632970 PMC3367386

[B14] Galluzzi L. Aaronson S. A. Abrams J. Alnemri E. S. Andrews D. W. Baehrecke E. H. (2009). Guidelines for the use and interpretation of assays for monitoring cell death in higher eukaryotes. *Cell Death Differ.* 16 1093–1107. 10.1038/cdd.2009.44 19373242 PMC2757140

[B15] Galluzzi L. Vitale I. Aaronson S. A. Abrams J. M. Adam D. Agostinis P. (2018). Molecular mechanisms of cell death: recommendations of the nomenclature committee on cell death 2018. *Cell Death Differ.* 25 486–541. 10.1038/s41418-017-0012-4 29362479 PMC5864239

[B16] Gao M. Monian P. Pan Q. Zhang W. Xiang J. Jiang X. (2016). Ferroptosis is an autophagic cell death process. *Cell Res.* 26 1021–1032. 10.1038/cr.2016.95 27514700 PMC5034113

[B17] Ginet V. Pittet M. P. Rummel C. Osterheld M. C. Meuli R. Clarke P. G. (2014). Dying neurons in thalamus of asphyxiated term newborns and rats are autophagic. *Ann. Neurol.* 76 695–711. 10.1002/ana.24257 25146903

[B18] Hamburger V. (1934). The effects of wing bud extirpation on the development of the central nervous system in chick embryos. *J. Exp. Zool.* 68 449–494. 10.1002/jez.1400680305

[B19] Hughes T. Rusten T. E. (2007). Origin and evolution of self-consumption: autophagy. *Adv. Exp. Med. Biol.* 607 111–118. 10.1007/978-0-387-74021-8_9 17977463

[B20] Julien O. Wells J. A. (2017). Caspases and their substrates. *Cell Death Differ.* 24 1380–1389. 10.1038/cdd.2017.44 28498362 PMC5520456

[B21] Jung S. Choe S. Woo H. Jeong H. An H. K. Moon H. (2020). Autophagic death of neural stem cells mediates chronic stress-induced decline of adult hippocampal neurogenesis and cognitive deficits. *Autophagy* 16 512–530. 10.1080/15548627.2019.1630222 31234698 PMC6999625

[B22] Kalkavan H. Green D. R. (2018). MOMP, cell suicide as a BCL-2 family business. *Cell Death Differ.* 25 46–55. 10.1038/cdd.2017.179 29053143 PMC5729535

[B23] Kanno H. Ozawa H. Sekiguchi A. Yamaya S. Itoi E. (2011). Induction of autophagy and autophagic cell death in damaged neural tissue after acute spinal cord injury in mice. *Spine* 36:1427. 10.1097/BRS.0b013e3182028c3a 21304420

[B24] Kosta A. Roisin-Bouffay C. Luciani M. F. Otto G. P. Kessin R. H. Golstein P. (2004). Autophagy gene disruption reveals a non-vacuolar cell death pathway in dictyostelium. *J. Biol. Chem.* 279 48404–48409. 10.1074/jbc.M408924200 15358773

[B25] Kroemer G. Levine B. (2008). Autophagic cell death: the story of a misnomer. *Nat. Rev. Mol. Cell. Biol.* 9 1004–1010. 10.1038/nrm2529 18971948 PMC2727358

[B26] Krysko D. V. Vanden Berghe T. D’Herde K. Vandenabeele P. (2008). Apoptosis and necrosis: detection, discrimination and phagocytosis. *Methods* 44 205–221. 10.1016/j.ymeth.2007.12.001 18314051

[B27] Levine J. S. Ucker D. S. (2019). Voices from the dead: the complex vocabulary and intricate grammar of dead cells. *Adv. Protein Chem. Struct. Biol.* 116 1–90. 10.1016/bs.apcsb.2019.02.004 31036289

[B28] Li H. Zhu H. Xu C. J. Yuan J. (1998). Cleavage of BID by caspase 8 mediates the mitochondrial damage in the fas pathway of apoptosis. *Cell* 94 491–501. 10.1016/s0092-8674(00)81590-1 9727492

[B29] Linder B. Kögel D. (2019). Autophagy in cancer cell death. *Biology* 8:82. 10.3390/biology8040082 31671879 PMC6956186

[B30] Lindqvist L. M. Simon A. K. Baehrecke E. H. (2015). Current questions and possible controversies in autophagy. *Cell Death Discov.* 1:15036. 10.1038/cddiscovery.2015.36 26682061 PMC4679147

[B31] Liu Y. Levine B. (2015). Autosis and autophagic cell death: the dark side of autophagy. *Cell Death Differ.* 22 367–376. 10.1038/cdd.2014.143 25257169 PMC4326571

[B32] Liu Y. Shoji-Kawata S. Sumpter R. M. Wei Y. Ginet V. Zhang L. (2013). Autosis is a Na+,K+-ATPase-regulated form of cell death triggered by autophagy-inducing peptides, starvation, and hypoxia-ischemia. *Proc. Natl. Acad. Sci. U. S. A.* 110 20364–20371. 10.1073/pnas.1319661110 24277826 PMC3870705

[B33] Lockshin R. A. Williams C. M. (1965). Programmed cell death–I. cytology of degeneration in the intersegmental muscles of the pernyi silkmoth. *J. Insect Physiol.* 11 123–133. 10.1016/0022-1910(65)90099-5 14287218

[B34] Lowe S. W. Schmitt E. M. Smith S. W. Osborne B. A. Jacks T. (1993). P53 is required for radiation-induced apoptosis in mouse thymocytes. *Nature* 362 847–849. 10.1038/362847a0 8479522

[B35] Malin J. Z. Shaham S. (2015). Cell death in C. elegans development. *Curr. Top. Dev. Biol.* 114 1–42. 10.1016/bs.ctdb.2015.07.018 26431562 PMC5206663

[B36] Martens S. Fracchiolla D. (2020). Activation and targeting of ATG8 protein lipidation. *Cell Discov.* 6:23. 10.1038/s41421-020-0155-1 32377373 PMC7198486

[B37] Martin D. N. Baehrecke E. H. (2004). Caspases function in autophagic programmed cell death in drosophila. *Development* 131 275–284. 10.1242/dev.00933 14668412

[B38] Nah J. Fernandez A. F. Kitsis R. N. Levine B. Sadoshima J. (2016). Does autophagy mediate cardiac myocyte death during stress? *Circ. Res.* 119 893–895. 10.1161/CIRCRESAHA.116.309765 27688304 PMC5161244

[B39] Nah J. Zhai P. Huang C. Y. Fernandez A. F. Mareedu S. Levine B. (2020). Upregulation of rubicon promotes autosis during myocardial ischemia/reperfusion injury. *J. Clin. Invest.* 130 2978–2991. 10.1172/JCI132366 32364533 PMC7260042

[B40] Nezis I. P. Shravage B. V. Sagona A. P. Johansen T. Baehrecke E. H. Stenmark H. (2010). Autophagy as a trigger for cell death: autophagic degradation of inhibitor of apoptosis dBruce controls DNA fragmentation during late oogenesis in drosophila. *Autophagy* 6 1214–1215. 10.4161/auto.6.8.13694 20935512 PMC3973654

[B41] Nirmala J. G. Lopus M. (2020). Cell death mechanisms in eukaryotes. *Cell Biol. Toxicol.* 36 145–164. 10.1007/s10565-019-09496-2 31820165

[B42] Nixon R. A. Wegiel J. Kumar A. Yu W. H. Peterhoff C. Cataldo A. (2005). Extensive involvement of autophagy in alzheimer disease: an immuno-electron microscopy study. *J. Neuropathol. Exp. Neurol.* 64 113–122. 10.1093/jnen/64.2.113 15751225

[B43] Ohsumi Y. J. W. (2014). Historical landmarks of autophagy research. *Cell Res.* 24 9–23. 10.1038/cr.2013.169 24366340 PMC3879711

[B44] Raff M. C. (1992). Social controls on cell survival and cell death. *Nature* 356 397–400. 10.1038/356397a0 1557121

[B45] Reed J. C. (2002). Apoptosis-based therapies. *Nat. Rev. Drug Discov.* 1 111–121. 10.1038/nrd726 12120092

[B46] Saunders J. W. Jr. Gasseling M. T. Saunders L. C. (1962). Cellular death in morphogenesis of the avian wing. *Dev. Biol.* 5 147–178. 10.1016/0012-1606(62)90008-8 14497532

[B47] Schweichel J. U. Merker H. J. (1973). The morphology of various types of cell death in prenatal tissues. *Teratology* 7 253–266. 10.1002/tera.1420070306 4807128

[B48] Shen H. M. Codogno P. (2011). Autophagic cell death: loch ness monster or endangered species? *Autophagy* 7 457–465. 10.4161/auto.7.5.14226 21150268

[B49] Shimizu S. Kanaseki T. Mizushima N. Mizuta T. Arakawa-Kobayashi S. Thompson C. B. (2004). Role of bcl-2 family proteins in a non-apoptotic programmed cell death dependent on autophagy genes. *Nat. Cell Biol.* 6 1221–1228. 10.1038/ncb1192 15558033

[B50] Smith K. B. Tata J. R. (1976). Cell death. are new proteins synthesized during hormone-induced tadpole tail regression? *Exp. Cell Res.* 100 129–146. 10.1016/0014-4827(76)90335-9 179829

[B51] Surh C. D. Sprent J. (1994). T-cell apoptosis detected in situ during positive and negative selection in the thymus. *Nature* 372 100–103. 10.1038/372100a0 7969401

[B52] Tang D. Kang R. Berghe T. V. Vandenabeele P. Kroemer G. (2019). The molecular machinery of regulated cell death. *Cell Res.* 29 347–364. 10.1038/s41422-019-0164-5 30948788 PMC6796845

[B53] Tsapras P. Nezis I. (2017). Caspase involvement in autophagy. *Cell Death Differ.* 24 1369–1379. 10.1038/cdd.2017.43 28574508 PMC5520455

[B54] Vakifahmetoglu-Norberg H. Xia H. G. Yuan J. (2015). Pharmacologic agents targeting autophagy. *J. Clin. Invest.* 125 5–13. 10.1172/JCI73937 25654545 PMC4382252

[B55] Vanden Berghe T. Linkermann A. Jouan-Lanhouet S. Walczak H. Vandenabeele P. (2014). Regulated necrosis: the expanding network of non-apoptotic cell death pathways. *Nat. Rev. Mol. Cell Biol.* 15 135–147. 10.1038/nrm3737 24452471

[B56] Wanderoy S. Hees J. T. Klesse R. Edlich F. Harbauer A. B. (2020). Kill one or kill the many: interplay between mitophagy and apoptosis. *Biol. Chem.* 402 73–88. 10.1515/hsz-2020-0231 33544491

[B57] Wang F. Gómez-Sintes R. Boya P. (2018). Lysosomal membrane permeabilization and cell death. *Traffic* 19 918–931. 10.1111/tra.12613 30125440

[B58] Wu M. Y. Yiang G. T. Liao W. T. Tsai A. P. Cheng Y. L. Cheng P. W. (2018). Current mechanistic concepts in ischemia and reperfusion injury. *Cell Physiol. Biochem.* 46 1650–1667. 10.1159/000489241 29694958

[B59] Xu H. D. Qin Z. H. (2019). Beclin 1, bcl-2 and autophagy. *Adv. Exp. Med. Biol.* 1206 109–126. 10.1007/978-981-15-0602-4_5 31776982

[B60] Yaoita Y. (2019). Tail resorption during metamorphosis in xenopus tadpoles. *Front. Endocrinol.* 10:143. 10.3389/fendo.2019.00143 30923513 PMC6426756

[B61] Yu L. Wan F. Dutta S. Welsh S. Liu Z. Freundt E. (2006). Autophagic programmed cell death by selective catalase degradation. *Proc. Natl. Acad. Sci. U. S. A.* 103 4952–4957. 10.1073/pnas.0511288103 16547133 PMC1458776

[B62] Yu S. W. Baek S. H. Brennan R. T. Bradley C. J. Park S. K. Lee Y. S. (2008). Autophagic death of adult hippocampal neural stem cells following insulin withdrawal. *Stem Cells* 26 2602–2610. 10.1634/stemcells.2008-0153 18653772

[B63] Zhao M. Chen J. Mao K. She H. Ren Y. Gui C. (2019). Mitochondrial calcium dysfunction contributes to autophagic cell death induced by MPP(+) via AMPK pathway. *Biochem. Biophys. Res. Commun.* 509 390–394. 10.1016/j.bbrc.2018.12.148 30594390

[B64] Zindel J. Kubes P. (2020). DAMPs, PAMPs, and LAMPs in immunity and sterile inflammation. *Annu. Rev. Pathol.* 15 493–518. 10.1146/annurev-pathmechdis-012419-032847 31675482

